# Maternity care in the Brussels Capital Region: Towards a paradigm shift?

**DOI:** 10.18332/ejm/186405

**Published:** 2024-05-21

**Authors:** Joeri Vermeulen, Maaike Fobelets, Aline Schoentjes, Laura Boucher, Laure Depuydt, Florence D’haenens

**Affiliations:** 1 Faculty of Medicine and Pharmacy, Department of Public Health, Biostatistics and Medical Informatics Research group, Vrije Universiteit Brussel, Brussels, Belgium; 2 Department of Health Care, Brussels Expertise Centre for Healthcare Innovation, Erasmus Brussels University of Applied Sciences and Arts, Brussels, Belgium; 3 Brussels Institute for Teacher Education, Vrije Universiteit Brussel, Brussels, Belgium; 4 Amala Espace Naissance, Brussels, Belgium; 5 Projet d’accompagnement des structures hospitalières et de première ligne dans l’implémentation de la réforme des normes hospitalières en région bruxelloise (Volet périnatal), Brussels, Belgium; 6 Department of Obstetrics and Gynecology, The Brussels University Hospital Brussels, Le Cocon, Brussels, Belgium; 7 Midwifery Department, Odisee University of Applied Sciences, Sint Niklaas, Belgium

**Keywords:** maternity care, Brussels capital region, midwifery units, midwifery-led care, women-centered care, autonomy

The shift towards midwifery-led care, and the recognition of midwives as autonomous primary health professionals reflects a global trend towards safe and quality women-centered care^1,2^. In Belgium, this trend was not apparent within the medicalized maternity care context^3^. To date, 98% of the births take place in a hospital setting, of which the majority are attended by an obstetrician. Nowadays, midwives have a decisive role, primarily in the ante- and post-natal period. The Common Community Commission, in charge with the policy on healthcare in Brussels, approved a revolutionary law of 25 May 2023, aiming to optimize perinatal care in the Brussels Capital Region^4^. The aim of the law is to promote both the accessibility and quality of welfare and care trajectories. Additionally, it seeks to redesign the welfare and healthcare landscape to provide integrated services in neighborhoods and to reposition the hospital within the broader care landscape. The law outlines the following specific regulations for maternity care: continuity of perinatal care, and postnatal follow-up at home and alongside midwifery units. Current developments not only signify an innovative approach to healthcare policy in Belgium, but could also serve as a model for improving maternal and infant health outcomes on an international scale^5^. Here, we emphasize the measures of the Brussels’ model related to guaranteeing accessibility of perinatal care and alongside midwifery units.

## Brussels’ maternity care model – history and current developments

Over the last decade, a shift in maternity care provision has been taken place in the Brussels Capital Region. Since the 1950s, Belgium has had an obstetrician-led care model for maternity care, with perinatal care predominantly provided by obstetricians. As an answer to this, a paradigm shift was initiated in 2014 in the Brussels Capital Region, by the establishment of the first alongside midwifery unit ‘Le Cocon’. A midwifery unit is defined as a location offering maternity care to healthy women with straightforward pregnancies in which midwives take primary professional responsibility for care. Midwifery units may be located adjacent to or alongside an obstetric service, or standing alone, freestanding from a hospital unit^6^. To this day, Le Cocon and Le Nid (established in 2019 in Namur) are the only alongside midwifery units in Belgium. These midwifery units combine the reassuring atmosphere of a home-like environment with the safety of an obstetrician-led unit nearby. Le Cocon is conceptualized as a low technicity midwifery unit^7^. It is managed solely by qualified midwives, with approximately 200 births annually (representing 11% of births in this hospital), guaranteeing one-to-one care. A recent study by Welffens et al.^8^ revealed that women planning their births in Le Cocon, experienced significantly lower cesarean births, inductions, epidurals, and episiotomies, with no increase in adverse neonatal outcomes. A retrospective Master’s study aimed to identify rates, reasons for, and risk factors of intrapartum transfers from Le Cocon to the obstetric unit, concluded that a significant proportion of transfers were driven by a desire for epidural analgesia, alongside issues related to labor progression^9^. These results are in line with international literature^10,11^. Additionally, a cost-effectiveness analysis study conducted at Erasme University Hospital, Brussels, suggests that incorporating midwifery units might imply a cost reduction for both women and the national payer; however, further research on larger sample sizes and from different health economic perspectives is needed to draw definitive conclusions regarding the cost-effectiveness of alongside midwifery units in Belgium^12^. The promising findings from these studies, along with comparable research, initially provided support for Brussels policymakers for the introduction of similar midwifery-led care pathways in other hospitals in the Brussels Capital Region^8^.

The law of May 2023, formalizes and stimulates the current shift in Brussels from obstetric-led to midwifery-led maternity care for straightforward pregnancies. From September 2024 on, Brussels hospitals with birthing facilities, subject to this particular legislation (n=8), will be required to integrate a midwifery birth unit, inspired by the Le Cocon model. While the practice of childbirth with the assistance of an obstetrician and medical staff is the norm in Belgium, the midwifery birth unit offers expectant mothers an alternative in the support of their pregnancy. This new approach, which will tend to become more widespread in Brussels in the coming months, will allow women who are uncomfortable with the traditional hospital setting to feel more secure with provided care. The reform will offer the expectant mother the choice of the setting for her child’s birth^13^.

This move is aligned with promoting informed decision-making and continuity of care. Any change in current maternity care is expected to be coupled with concrete structural changes as well as cultural changes^14^. The reorganization of maternity care is expected to be accompanied with strategies to increase public awareness of the particular role of midwives in the continuum of care, for both women and their families. However, the project currently faces a significant obstacle as there is a lack of a comprehensive plan and necessary budget to enhance awareness. Involvement of all concerned, women’s groups, other maternity care professionals and policymakers, is key to initiate this structural change^15^. However, with the implementation of the Brussels’ maternity care model in just a few months, concerns arise regarding the adequate remuneration of self-employed, primary care midwives and the readiness of the healthcare system to accommodate this change. Specifically, midwives need to be adequately prepared to staff these birth centers, which will be crucial for the success of this initiative. To address this challenge, efforts are being made to provide postgraduate training opportunities for midwives to enhance their skills in supporting straightforward pregnancies and births, and managing emergencies.

For hospitals who do not manage to organize an alongside midwifery unit, hospitals are obliged to allow primary care professionals, midwives and general practitioners, in the hospital to accommodate parents who wish to give birth naturally, feel at home, while being close to medical interventions if needed. The current situation in Belgium is that primary care professionals have limited access to hospitals^15^, as a structured collaboration between midwives and hospitals is generally restricted to postpartum home care^16^. The number of maternity facilities, open to primary care midwives^17,18^, range from 45.5% of the hospitals in Brussels to 6–10% in the other Belgian regions. Nevertheless, the recent Brussels’ law is strongly backed by recent research, indicating that enhancing the conditions for primary care midwives to gain access to hospitals in order to promote midwifery-led continuity of care models contributes to safe and qualitative care for all service-users^15^. Additionally, the Flemish Professional Association of Midwives has recently proposed a model aiming to support midwifery-led birth guidance as full-fledged option within the current traditional maternity care in Belgium. This publication establishes a well-founded basis for successful collaboration between primary care midwives seeking to autonomously assist births in the hospital^17^. The recent Brussels’ law encourages hospitals to provide birthing facilities for primary care professionals through agreements that outline financial terms, responsibilities, capacity, timelines, and information exchange. The law additionally aims to ensure quality maternity care by promoting collaboration between healthcare professionals to ensure the transmission of essential information between primary care professionals and hospitals. Additionally, agreements must be made between hospitals and midwives for postnatal follow-up at home, with specific details regarding capacity, timelines, information exchange, and fees. The aforementioned set of innovative measures aims to facilitate access of primary care professionals to hospitals, strengthen coordination between hospitals and primary care midwives, and ensuring comprehensive care surrounding childbirth.

## Towards a bright future

The shift towards midwifery-led care represents a potential turning point in Belgian birthing culture, offering women, families and midwives more autonomy and options for their birthing experience. The successful initiative of Le Cocon, together with other local initiatives of primary care midwives, led to new insights regarding the organization of perinatal care in Brussels. As in other countries, the implementation of the Brussels’ maternity care model holds promise for ensuring safe and high-quality women-centered care. This approach guarantees the accessibility of perinatal care and alongside midwifery units, all without a rise in maternal or neonatal complications, as evidenced by recent studies^19,20^. Furthermore, researchers are challenged to evaluate this subject to further enhance understanding and implementation. The existing evidence highlighting the advantages of continuity of perinatal care and alongside midwifery units, and the growing interest in different childbirth care models, may inspire all Belgian regional governments to integrate the Brussels’ maternity care model into their healthcare systems. In addition, the Brussels’ model may serve as inspiration for countries encountering similar challenges, prompting them to explore its implementation and potential benefits within their own healthcare structures.

## Data Availability

Data sharing is not applicable to this article as no new data were created.
