# Corrigendum: Maternity care in the Brussels Capital Region: Towards a paradigm shift?

**DOI:** 10.18332/ejm/189477

**Published:** 2024-06-05

**Authors:** Joeri Vermeulen, Maaike Fobelets, Aline Schoentjes, Laura Boucher, Laure Depuydt, Florence D’haenens

**Affiliations:** 1 Faculty of Medicine and Pharmacy, Department of Public Health, Biostatistics and Medical Informatics Research group, Vrije Universiteit Brussel, Brussels, Belgium; 2 Department of Health Care, Brussels Expertise Centre for Healthcare Innovation, Erasmus Brussels University of Applied Sciences and Arts, Brussels, Belgium; 3 Brussels Institute for Teacher Education, Vrije Universiteit Brussel, Brussels, Belgium; 4 Amala Espace Naissance, Brussels, Belgium; 5 Projet d’accompagnement des structures hospitalières et de première ligne dans l’implémentation de la réforme des normes hospitalières en région bruxelloise (Volet périnatal), Brussels, Belgium; 6 Department of Obstetrics and Gynecology, The Brussels University Hospital Brussels, Le Cocon, Brussels, Belgium; 7 Midwifery Department, Odisee University of Applied Sciences, Sint Niklaas, Belgium

**Keywords:** maternity care, Brussels capital region, midwifery units, midwifery-led care, women-centered care, autonomy

Corrigendum on:

Maternity care in the Brussels Capital Region: Towards a paradigm shift?

By Joeri Vermeulen, Maaike Fobelets, Aline Schoentjes, Laura Boucher, Laure Depuydt, Florence D’haenens

European Journal of Midwifery, Volume 8, Issue May, Pages 1-4.

Publish date: 21 May 2024

DOI: https://doi.org/10.18332/ejm/186405

In the originally published version of the article, there was a Table that now has been removed. This is corrected also online.

